# 3D printing of pharmaceuticals for disease treatment

**DOI:** 10.3389/fmedt.2022.1040052

**Published:** 2023-01-10

**Authors:** L. R. Jaidev Chakka, Shanthi Chede

**Affiliations:** ^1^ College of Pharmacy, The University of Texas at Austin, Austin, TX, United States; ^2^ College of Pharmacy, University of Iowa, Iowa, IA, United States

**Keywords:** 3D printing (3DP), cancer, implants — artificial, pharmaceuical, buccal activity

## Abstract

Three-dimensional (3D) printing or Additive manufacturing has paved the way for developing and manufacturing pharmaceuticals in a personalized manner for patients with high volume and rare diseases. The traditional pharmaceutical manufacturing process involves the utilization of various excipients to facilitate the stages of blending, mixing, pressing, releasing, and packaging. In some cases, these excipients cause serious side effects to the patients. The 3D printing of pharmaceutical manufacturing avoids the need for excessive excipients. The two major components of a 3D printed tablet or dosage form are polymer matrix and drug component alone. Hence the usage of the 3D printed dosage forms for disease treatment will avoid unwanted side effects and provide higher therapeutic efficacy. With respect to the benefits of the 3D printed pharmaceuticals, the present review was constructed by discussing the role of 3D printing in producing formulations of various dosage forms such as fast and slow releasing, buccal delivery, and localized delivery. The dosage forms are polymeric tablets, nanoparticles, scaffolds, and films employed for treating different diseases.

## Introduction

**Figure 1:**
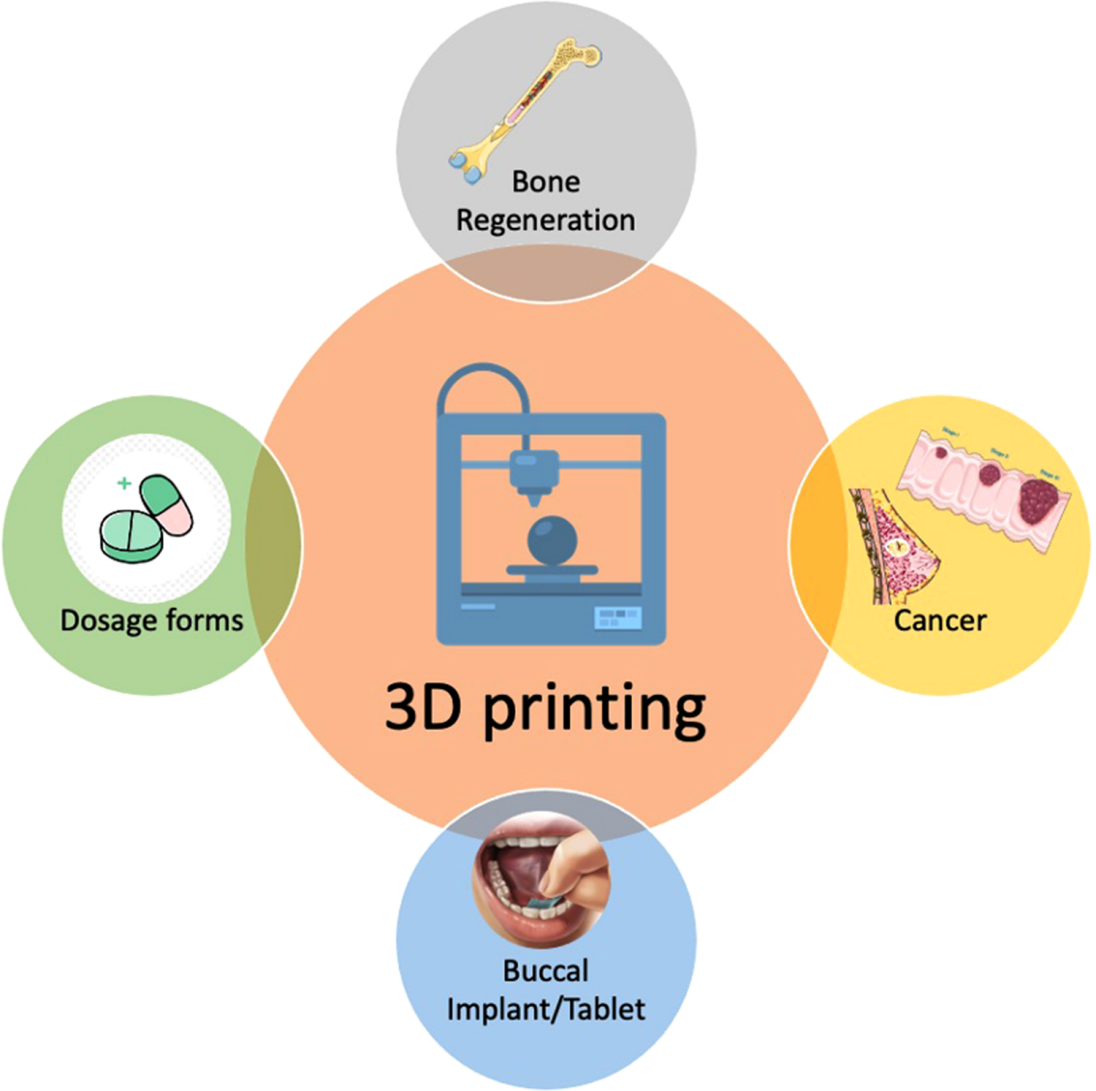
Cartoon showing the applications of 3D printing in disease treatment

Three-dimensional (3D) printing has progressed the stagnant pharmaceutical industry to develop medical dosages beyond earth and space (1). Compared to traditional manufacturing methods, such as compacting, milling, and molding, 3D printing offers many advantages such as rapid and on-demand production, flexibility in terms of drug dosages, and complexity in terms of object geometry (2, 3). The tailoring of drug combinations and concentrations to individual requirements and the shape of any printed devices to specifically match one's anatomy (4). Researchers expressed their views regarding the stages of improvement needed for developing pharmaceuticals with 3D printing (5). The interest of healthcare professionals has increased by more than 60% for prescribing 3D printed tablets (6). In general, 3D printing has been used but not limited to bone tissue regeneration and implants for disease treatments such as cancer, oral dosage forms and buccal implants (Figure 1).

Technological advancements have changed the face of the earth with the first and second industrial revolutions. The rapid development of 3D printing and its application in product manufacturing has been considered the third industrial revolution. These advancements can ease a similar impact on the pharmaceutical industry. The pharmaceutical industry is very conserved for several decades if not centuries. The industry typically follows the established manufacturing processes to make dosage forms such as tablets, capsules, etc. With the power of novel technological advancements, the industry can revolutionize the way medicines are designed and manufactured. The three-dimensional (3D) printing technology has evolved from a do-it-yourself rudimentary stage to the highly advanced state-of-the-art developed stage. Established companies such as Stratasys, 3D systems, etc., are developing new and advanced 3D printers for manufacturing auto and aero parts. However, the adaptation of this technology in the pharma industry is still in its nascent stage. There are no major players in the pharma industry developing pharmaceuticals for everyday use. There are several startups like Teva and Aprecia pharmaceuticals that are exploring this opportunity. Aprecia Pharmaceuticals is the first company that produced and marketed the first FDA-approved 3D printed formulation called “Spritam” (Levetiracetam) for epilepsy in 2015 using their patented Zip dose technology (5).

## Types of 3D printing

The technology differs based on the technique used to achieve the 3D shape. Several 3D printing methods are being explored by many researchers around the world. Fused Deposition Modeling, Extrusion based printing, Selective Laser ablation, Stereolithography, Binder jet printing, and powder-bed method are the widely used 3D printing methods across different disciplines. Each technique has its unique advantages owing to the kind of material and the field of application.

Fused Deposition Modeling (FDM) is an extremely popular 3D printing technique that is widely employed for manufacturing a wide variety of products. The adaptation of this technique in tablet manufacturing has opened new venues for the design of unique formulations (7). The hot-melt extrusion coupled dual nozzle printing of an FDM process delivered bilayer tablets for independent drug release profiles (8). Fixed-dose combinations are the new kind of formulations that achieve optimal goals for the treatment of disease with minimal side effects resulting in improved patient compliance (9). Chewable theophylline tablets for veterinary purposes were evaluated to meet an unmet need for precise off-label use of non-human products (10). Drug-loaded 3D films were developed for understanding the patient-centric effect of the drug release phenomenon (11). Recently, mathematical adaptations were employed to develop drug-loaded formulations to facilitate an optimized polymer and drug wt. percentages for a tailored drug release (12). Antibiotics are key for treating bacterial infections. However, the high dosage of antibiotics supplies bacterial resistance. To avoid that, consistent release of antibiotics avoids drug resistance. The 3D printed thermoplastic gentamycin-released PCL formulations avoided the growth of *S. aureus* in mice (13). The Key advantage is that the formulation development is faster, and the printing technology is well established giving it an easy fabrication process facilitating rapid production and scale up production lines. The major disadvantage of FDM is the high processing temperature not suitable for certain temperature sensitive drugs and biologics. Compatibility and stability are two important aspects of tablet evaluation for safety and efficacy (14). Semisolid extrusion is a 3D printing method carried out at room temperature that produces objects or devices out of gels or pastes. The re-dispersible formulations contain drug-loaded polymeric nano capsules inside a hydrogel for drug delivery applications (15). Effervescent tablets are another kind of medication that delivers the drug. The major advantage is that the formulation typically includes drug, acid, and alkali pastes which are printed in a localized manner to prevent any premature effervescence (16). The adaptability of the technology is still nascent in the pharmaceutical industry but highly popular with bioprinting for tissue regeneration applications. Selective laser sintering is another popular 3D printing technique used to prepare formulations and personalized implants. High volume of printing tablets can be carried out using this technique without the influence of personnel errors. The laser beam sinters a layer of the powder formulation which coalesce the particles together. The material with absorption of laser is key for improved resolution and tailoring drug release. This technique is being widely explored to print oral formulations (17–19). Blending of different drugs is very difficult with SLS. Binder jet printing is one of the earliest techniques that is used for automotive industry by has limited applications for drug delivery applications. Lu et al. has published a modular approach for releasing multiple drugs of anti-viral nature at a time. This work is very interesting and provided new ways of employing binder jet technology for pharmaceutical development (20). The binder jet printing is similar to selective laser sintering, but a high-resolution print head will deposit binder solution on to the powder bed resulting in a hardened 3D structure. However, this technology was adapted by Aprecia Pharmaceuticals and developed a revolutionary Zipdose technology and eventually succeed in launching Spritam (Levetiracetam) an Food and Drug Administration approved drug first time in the world. Stereolithography (SLA) has recently been used to fabricate tablets. SLA is also known as vat photopolymerization where the resin is hardened by the exposure to ultraviolet light resulting in a hardened structure. The resins used in this technique are highly toxic. The adaptation of existing resins for biomedical and pharmaceutical applications is a major limitation of this technique. However, recent reports showed the usage of biocompatible polytheyleneglycol diacrylate or other acrylates for tablet fabrication. The technology has a limitation of using only few materials that are biocompatible and UV curable. Development of new materials in future can accelerate the adaptation of this technology. Similarly, Immediate release tablets were fabricated using this technique to release Zolpidem tartrate (21). The same technique was also used to develop medical devices that have photothermal and shape memory functions (22).

The workflow of three-dimensional printing is the deposition, polymerization, or binding of the material of choice in a layer-by-layer fashion resulting in a final three-dimensional structure. The general process is identified as ‘3 Ds of 3D printing. These are (i) Design, (ii) Develop, and (iii) Dispense. Any 3D printing process start with the development of a computer-generated 3D model using software called computer-aided design (CAD). The model is a 3D projection with defined parameters based on the type of structure. These pre-defined parameters of the shape will be reflected in the final 3D printed structure. This CAD is exported as the widely popular.stl file extension. The slicing software will read this.stl file and process it into several layers and convert this information into a.gcode file. Depending on the printer type and the CAD design employed, the commands and information will be different, but all 3D printers, in general, can only read this .gcode file format. This stage is to identify the method for the development of dosage forms using the 3D printing technique, print parameters, suitable materials, and drugs to work with for a certain application. The users must ensure material compatibility, drug compatibility, and material-drug interactions based on the API properties to achieve an active, desirable dosage form. The 3D printer can then be filled with the drug-loaded feedstock. Formulations are prepared in a layer-by-layer fashion, which is then ready for “dispensing”. This method of production varies depending on the printing platform selected. This process can benefit industries and researchers to produce dosage forms for several clinical applications and a greater advancement in creating personalized medicines.

## 3D Printing pharmaceutics

### Fast releasing tablets

The fused deposition modeling (FDM) 3D printing technique has considerable potential for patient-specific dosage forms. Design approach where the caplets with perforated channels accelerate drug release from 3D printed tablets (23). The fabrication of ready-to-use immediate-release tablets *via* 3D printing provides a powerful tool for on-demand individualization of the dosage form. Fused deposition modeling (FDM) involves passing a filament based on thermoplastic polymers through a hot nozzle, where the temperature is elevated above its glass transition temperature (Tg). The major limitation of FDM 3D printing renders it unsuitable for the production of immediate-release tablets, which count for approximately 70% of all oral dosage forms (24). Alhan's group reported the fabrication of immediate-release tablet based on positively charged methacrylic polymers (25). Same group tested the release of Theophylline and Dipyridamole using polyvinylpyrrolidone polymer delivering a 100% release in 70 min (26).

### Buccal formulations

The 3D printing of oral formulations has been the most extensively investigated. Drug delivery across buccal mucosa is one of the promising alternative delivery approaches to deliver drug compounds that are degraded either due to high first-pass metabolism or due to harsh conditions in the GI tract. Considering the rich vasculature of buccal mucosa, the drug compounds can be directly delivered into the systemic circulation. In specific, buccal and sublingual mucosa of the oral cavity have been studied to be the most efficient and alternative delivery route for several drug compounds for both systemic and local delivery.

Thin-film systems constituting both mucoadhesive and or dispersible thin films are the most effective dosage forms suitable for oral mucosal administration. Currently, solvent casting technique and hot-melt extrusion are the most commonly used techniques for the preparation of oro-mucosal films or patches. However, several limitations have been identified for each of these processes including (1) long processing and drying times due to solvent evaporation for solvent casting technique. Also, solvent casting techniques are not suitable for APIs that are prone to hydrolysis (27), (2) hot-melt extrusion could improve the solubility of the API but is not suitable for thermo-labile drugs (27, 28). They also provide limited opportunities for patient-tailored dosing regimens and the incorporation of multiple functional materials targeting different drug release characteristics. Recently, 3D printing technology has been identified to be a successful approach for the design and development of various dosage forms intended for buccal administration. More specifically, 3D printing manufacturing techniques are very promising to develop personalized doses/various combinations, and films with robust mechanical and drug release characteristics (29, 30). Various 3D printing technologies have been evaluated for the manufacture of oro-mucosal films (31, 32) including fused deposition modeling (FDM) (33), inkjet printing (34), flexographic printing (35), and direct ink writing (robocasting/pressure-assisted 3D printing) (36). Below details summarize the recent studies that have evaluated various 3D printing technologies for the fabrication of buccal dosage forms.

### Buccal films

Mucoadhesive buccal films are one of the attractive approaches considering their enhanced muco-retention properties along with the permeation-enhancing ability for either local or systemic delivery options. Mucoadhesive films involve the usage of various functional excipients including mucoadhesive agents, permeation enhancers, and film formers attached to different backing layers. The drug compound is incorporated in different regions of the film based on the desired drug release characteristics. Spatial distribution of these components becomes important in designing buccal thin films since the functionality of these excipients plays a critical role in defining attachment of the film based on the site of administration, the direction of drug release, and the rate of drug release, followed by absorption of drug compounds across the buccal mucosa. In recent work, the effect of FDM, 3D printing technology was used in the manufacturing of Diclofenac Sodium multi-layered mucoadhesive layers (33). Their studies used poly(vinyl alcohol) (mucoadhesive) chitosan (mucoadhesive and permeation enhancer) along with ethyl cellulose and other commonly used commercial wafers as backing layers. Their results showed uniform content across all the 6 different formulation types indicating the FDM printing process was reproducible. Based on the SEM observations, the filament diameter was adjusted in the 3D printer software, resulting in avoiding excessive molten blend and producing buccal films that are more homogenous with a smooth external surface and no internal porous structures. Tensile strength measurements showed that backing layers exhibited poor mechanical properties. However, without backing layers, the flexibility of the 3D printed films tested by folding endurance resulted in a folding endurance of at least 300 times without breakage. Other *in vitro* testing also showed superior film properties including, unidirectional release and similar drug release behavior up to first 15 min from formulations loaded with either ethyl cellulose or wafer-containing backing layers. Chitosan-loaded films exhibited superior mucoadhesive strength (∼52%) and higher permeation-enhancing ability (>3 fold) across the porcine buccal mucosa compared to other formulations. Overall, their studies indicated that 3D printing of mucoadhesive buccal films produced acceptable film-forming properties with sufficient mechanical properties that can withstand the force at the site of administration (33). Also, the authors concluded that 3D printing could be a versatile approach for the manufacture of multilayered buccal films. A combination of FDM and ink-jet printing was investigated as an alternative manufacturing process in order to overcome the temperature-sensitive limitations of the FDM technique. Dual drug-loaded films were fabricated by utilizing both methods. FDM was used to fabricate HPMC-based mucoadhesive films loaded with NSAID (ketoprofen) and ink jet printing was used for the deposition of Lidocaine Hcl (LIH) along with l-menthol (permeation enhancer) on the film (37). Inkjet printing generated optimal design with an 80:20 v/v sample loaded with 300 mg/mL of Lidocaine HCL. SEM images showed the presence of some crystalline matter on the 3D printed HPMC substrates and ink-jet printed films. Based on DSC studies, an endotherm at 165°C was identified, which references plain HPMC, indicating that the recrystallization could be attributed to raw HPMC material. XRD studies showed few characteristic peaks that are representative of HPMC, LiH, and menthol indicating the existence of crystalline forms of both the polymer and LiH on the ink-jet printed film systems. Even though ink deposition effected onto the mucoadhesive HPMC substrate resulted in some alterations in the overall morphology of the films, no change in drug release characteristics or drug absorption across the buccal epithelial cell layers (both porcine mucosa and TR146 cell layers) was observed. Overall, this study demonstrated proof of concept of the use of the FDM/ink-jet dual printing process for the incorporation of temperature-sensitive drug compounds in mucoadhesive films (37). Another recent study demonstrated the use of direct ink writing (DIW) technology for the fabrication of more complex and unique design features containing saquinavir (antiretroviral drug compound) with pH_m_ (microenvironment) modifying buccal patches. This study used the integration of three inks on a sub-millimeter scale (38). Printing materials include acidic saquinavir-loaded HPMC ink and alkaline sodium-carbonate ink incorporated on a methylcellulose backing layer resulting in buccal patches with a mesh design that exhibited superior stretchable mechanical properties (38).

### Oral dispensable films using 3D printing

Oral dispensable films are thin polymeric systems that are dissolved readily within the oral cavity. Oro dispersible films rapidly disintegrate in presence of saliva, and the active drug compounds released are either absorbed through the mucosal route within the oral cavity or ingested through saliva into the gastrointestinal tract. FDM was first explored to evaluate Aripriprazole-PVA-based dispersible films (39). 3D printed films were compared to the films prepared by solvent casting technique for physical and mechanical properties. DSC and XRD studies confirmed that the 3D printing process has transitioned the crystalline nature of Aripiprazole to the amorphous state resulting in an increase in dissolution rate where 3D printed films released 95% Aripiprazole within 15 min whereas, 75% of aripiprazole was released within 15 min from solvent cast films (39).

### Transdermal drug delivery applications

3D printing has been identified as a successful approach for the fabrication of pharmaceutical drug product dosage forms of various geometries for transdermal delivery applications. Based on the therapeutic requirement, 3D printing was successfully used for the development of several dosage forms like microneedles, patches, and implants for both systemic and local delivery of drug compounds. In one study, Allen et al. piezoelectric inkjet printing process was used for the fabrication of vaccine-loaded dissolvable microneedles (40). They were fabricated by dispensing liquid formulation using the drop-on-drop deposition technique onto Polydimethylsiloxane (PDMS) microneedle molds. As a part of their study, it was observed that the stable precise piezoelectric dispensing process was primarily dependent on formulation attributes (viscosity, wettability) and actuation setting. Their initial design of experiments demonstrated that a 1% PVA-based formulation with a viscosity of 4–8 cP generated a more precise piezoelectric dispensing system with optimal PDMS mold wetting (contact angle <100°) and stable drop formation. After dispensing, the biological integrity of the vaccine was tested at a range of voltages (30 V, 50 V, and 80 V) and it was observed that the biological integrity of the vaccine was maintained only low voltage setting (30 V). Overall, their results demonstrated successful usage of the piezoelectric inkjet 3D printing technique for the fabrication of vaccine-loaded microneedle systems. Another study also used the inkjet printing technique to coat the 3D microneedle arrays with three anticancer components including curcumin, cisplatin, and 5-fluorouracil, for transdermal drug delivery (41). It was reported that the piezoelectric dispensing technique produced uniform and reproducible drug coating on the microneedles. In another approach, Economidou et al. prepared 3D printed microneedle arrays using stereolithography technique containing biocompatible resin for insulin delivery *via* transdermal application. It has been identified that a uniform printing process developed microneedles with enhanced penetration capacity with minimally applied forces (2–5 N). PK studies have also demonstrated faster insulin action resulting in adequate hypoglycemic action within 60 min (42).

### 3D printable implants

3D printable drug eluting implants has received considerable attention due to various printing options, biocompatible printing materials and several customized and personalized implants. 3D printed implants for several cancer categories have been evaluated, and few of them are presented in Table 1. Few Studies showed the application of 3D printed implant for cancer immunotherapy (43, 44). Mao et al., developed a 3D printed nanogel implant for the eradication of residual glioblastoma cells from the tumor cavities after the surgery. In their study, 3D printed therapeutic device was engineered that could match the post-surgery tumor cavity and release oncolytic DNA nanocomplexes to kill glioblastoma cells. In order to test this, 3D printed implant was inserted into a subcutaneous glioblastoma xenograft and identified that the release of DNA nanocomplexes resulted in a significant delay of glioblastoma cells and efficiently delays the tumor recurrence. Thus, this study provided a proof- of concept of 3D printed Implant delivery system for glioblastoma therapy (43). Culp et al. recently demonstrated the therapeutic efficacy of microsphere eluting polymeric implant for hepatocellular neoplasia (45). A standard chemotherpay treatment involve multiple drugs. Tsai et al. developed an implantable microdevice to deliver 12 chemotherapeutic drugs to patients for treating non-small cell lung carcinoma (46).

**Table 1:** Delivery systems overview.

Drug	Polymer	3D printing method	Delivery target	Dosage Form	Drug release duration	Disease	Ref.
Decarbazine	Poly (propylene fumarate) and diethyl fumarate (DEF)	Microstereolithography	N/A	Microneedle	5 weeks	Skin Cancer	(47)
Cisplatin	Soluplus® (co-polymer of polyvinyl caprolactame-polyvinyl acetate-polyethylene glycol	Stereolithography, inkjet printing	Balb/c nude mice	Microneedle	Rapid release	Skin Cancer	(27)
Doxycycline	Mesoporous bioactive glass/polycaprolactone (Fe_3_O_4_/MBG/PCL)	3D Scaffolds printed by 3D Bioplotter™	Primary human bone marrow-derived mesenchymal stem cells (h-BMSCs)	Magnetic hyperthermia 3D Scaffolds	Sustained Release for 9 days	Bone Cancer	(28)
	Ca-P/polydopamine		rabbit bone mesenchymal stem cells (rBMSCs)	Photothermal bioscaffold		Bone Cancer	(29)
Methylene Blue, Docetaxel	PDMS (polydimethylsiloxane)		*ex vivo* porcine bladder tissue	Cylindrical magnetically-actuated Implant		Prostrate cancer	(30)
Doxycycline, Ifosfamide, Methotrexate, Cisplatin	Poly L-lactic acid	stereolithography	Human osteosarcoma U2OS cells	Spherical Implant	Greater than 12 weeks	Osteosarcoma	(31)
Doxcycline and Cisplatin	Poly-lactic-co-glycolic acid (PLGA)	E-jet 3D printing	MDA-MB-231 cells	Scaffold		Breast cancer	(32)
Paclitaxel, Rapamycin	Poloxamer 407	3D printing by fused deposition technology	Intraperitoneal delivery in ES-2-luc ovarian-cancer-bearing xenograft mice	Nanogel discs	Not less than 6 h	Ovarian cancer	(33)

### 3D printing for other local delivery applications

3D printing technologies have been used for several local delivery applications. A few of them are summarized here. In one study, Goyanes et al. designed and developed a personalized nose mask that can deliver anti-acne drug (salicylic acid) by topical delivery. 3D model of a nose scan of the person was obtained from 3D scanning technology and a nose mask was fabricated using Flex EcoPLA™ (FPLA) and polycaprolactone (PCL) polymer material incorporated with salicylic acid by FDM hot-melt extrusion (HME) technique. The *in vitro* diffusion test data showed that a 3D printed nose mask released <187 μg/cm^2^ of salicylic acid within 3 h (48). Another study showed the usage of FMD-based 3D printing techniques to develop patient-specific anti-microbial wound dressings (49). This study developed various antimicrobial wound dressings with different shapes were generated using an FDA approved polymer (polycaprolactone- PCL) filaments incorporated with various concentrations of metals (Ag (10% w/w)-PCL, Zn (10% w/w)-PCL, Zn (25% w/w)-PCL, Cu (10% w/w)-PCL and Cu (25% w/w)-PCL)). It was observed that the fabricated wound dressings showed fast drug release (up to 24 h) followed by slow release (up to 72 h). Overall, their study demonstrated that personalized customizable wound dressings with respect to shape, size, and type of antimicrobial agents can be developed using 3D printing techniques. Another study demonstrated the usage of a 3D printed biodegradable patch for local delivery of 5-fluorouracil at the tumor site (50). Polycaprolactone and poly (lactic-co-glycolic acid) were used as polymeric compounds and fabricated systems exhibited prolonged drug release characteristics for up to 4 weeks. It was also understood that the drug release properties were highly dependent on the size of the patch. Thus, the results conclude that 3D printing could be a promising approach for local delivery applications.

## Future directions

Deviating conventional pharmaceutical manufacturing has opened all new possibilities for exploring the development of dosage forms in terms of design, materials, and processing. The advancements in 3D printing of pharmaceuticals are progressed towards realizing the end goal of personalized medicine with on-demand manufacturing capabilities. Aprecia Pharmaceuticals is partnering with Cycle Pharmaceuticals to print other approved, orphan drugs using this technology. The regulatory changes for 3D printing technology is being actively undergoing in Food and Drug Administration and everal guidelines were published in their website. The inception of new Good Manufacturing Practices grade 3D printers might be a key in unleashing the potential of the 3D printing technology as a mainstream tablet manufacturing in pharma industry.
